# Hepatocyte-targeting and tumor microenvironment-responsive liposomes for enhanced anti-hepatocarcinoma efficacy

**DOI:** 10.1080/10717544.2022.2122635

**Published:** 2022-09-14

**Authors:** Dongliang Cheng, Zhiwei Wen, Hui Chen, Shiyuan Lin, Wei Zhang, Xin Tang, Wei Wu

**Affiliations:** a School of Pharmacy, Guilin Medical University, Guilin, China; b School of Chinese Materia Medica, Guangzhou University of Chinese Medicine, Guangzhou, China; c School of Public Health, Guilin Medical University, Guilin, China

**Keywords:** Sodium taurocholate cotransporting polypeptide, matrix metalloproteinase-2, enzyme sensitive liposomes, liver targeting, hydroxycamptothecin

## Abstract

To increase the antitumor drug concentration in the liver tumor site and improve the therapeutic effects, a functionalized liposome (PPP-LIP) with tumor targetability and enhanced internalization after matrix metalloproteinase-2 (MMP2)-triggered cell-penetrating peptide (TATp) exposure was modified with myrcludex B (a synthetic HBV preS-derived lipopeptide endowed with compelling liver tropism) for liver tumor-specific delivery. After intravenous administration, PPP-LIP was mediated by myrcludex B to reach the hepatocyte surface. The MMP2-overexpressing tumor microenvironment deprotected PEG, exposing it to TATp, facilitating tumor penetration and subsequent efficient destruction of tumor cells. In live imaging of small animals and cellular uptake, PPP-LIP was taken up much more than typical unmodified liposomes in the ICR mouse liver and liver tumor cells. Hydroxycamptothecin (HCPT)-loaded PPP-LIP showed a better antitumor effect than commercially available HCPT injections among MTT, three-dimensional (3 D) tumor ball, and tumor-bearing nude mouse experiments. Our findings indicated that PPP-LIP nanocarriers could be a promising tumor-targeted medication delivery strategy for treating liver cancers with elevated MMP2 expression.

## 1. Introduction

Hepatocellular carcinoma (HCC) is a common malignancy that is very aggressive in the clinic, and it is often fatal. Chemotherapy has long been the treatment of choice for liver cancer patients because of the poor clinical outcomes of radiation and surgery (Zheng et al., 2019; Huang et al., 2021). The applications of pharmaceutical nano-carriers to improve the precise tumor delivery of anticancer medications have been well known, several ways for delivering therapeutic drugs to liver cancer patients have been devised (Russell et al., 2020; Wu et al., 2020). Using a size-dependent passive targeting technique, liposomes and polymeric nanoparticle delivery systems, such as Doxil®, doxorubicin hydrochloride liposome injection (Barenholz, 2012), have been widely explored as liver-specific drug carriers.

Conventional liposomes surface modified with targeting ligands (e.g. peptides, antibodies, or aptamers) have higher drug loading, targeted drug delivery and enhanced anticancer activity (Guan et al., 2018; Allemailem, 2021). Sodium taurocholate co-transporting polypeptide (NTCP) is a functional receptor for hepatitis B virus (HBV) entry in liver cells (Yan et al., 2012). Myrcludex B (myrB), HBV preS/12-47myr, is a lipopeptide generated from the HBV L-protein that can recognize and bind to NTCP (Cheng et al., 2021). HBV preS-derived lipopeptide, HBV preS/2-48myr, was conjugated to PEGylated liposomes to enhance liver-specific delivery through an NTCP receptor-mediated endocytosis mechanism (Zhang et al., 2014b). Liver-targeted PEGylated liposomes based on hepatitis B virus’s N-terminal myristoylated preS1/21-47 (preS1/21-47myr) were prepared, which revealed significant liver-specific transport and an increase in the distribution of preS1/21-47myr-PEG-LIP in hepatic tumors (Zhang et al., 2015). Moreover, in order to enhance the penetration of targeting nanoparticles into liver tumor cells, cell-penetrating peptides (such as TATp) were utilized to boost the uptake of the nanomedicines (Jones & Sayers, 2012; Pan et al., 2012; Gao et al., 2014; Hu et al., 2021).

Polyethylene glycol (PEG)-phospholipids linked to peptide ligands are widely used for liposomes surface modification. However, the PEG chains may obstruct liposome entry into deep tumors and internalization into cells, resulting in diminished anticancer efficacy (Bao et al., 2016; Hou et al., 2016), deprotection of PEG chains in tumor areas is critical for cancer treatment efficacy (Ma et al., 2018). Matrix metalloproteinases-2 (MMP2) is implicated in the invasion, development, and metastasis of the majority of human cancers due to their propensity to degrade the surrounding connective extracellular matrix (ECM). MMP2 is overexpressed in malignancies such as breast, colorectal, lung, liver, prostate, pancreatic, and ovarian (Kessenbrock et al., 2010). MMP2-cleavable peptide (GPLGIAGQ) was employed to initiate the de-shielding of liposomal carriers from PEG, resulting in increased cellular internalization and enhanced drug distribution in the tumor area (Fang et al., 2011; Bertrand et al., 2014; Yao et al., 2018).

**Figure 1.**
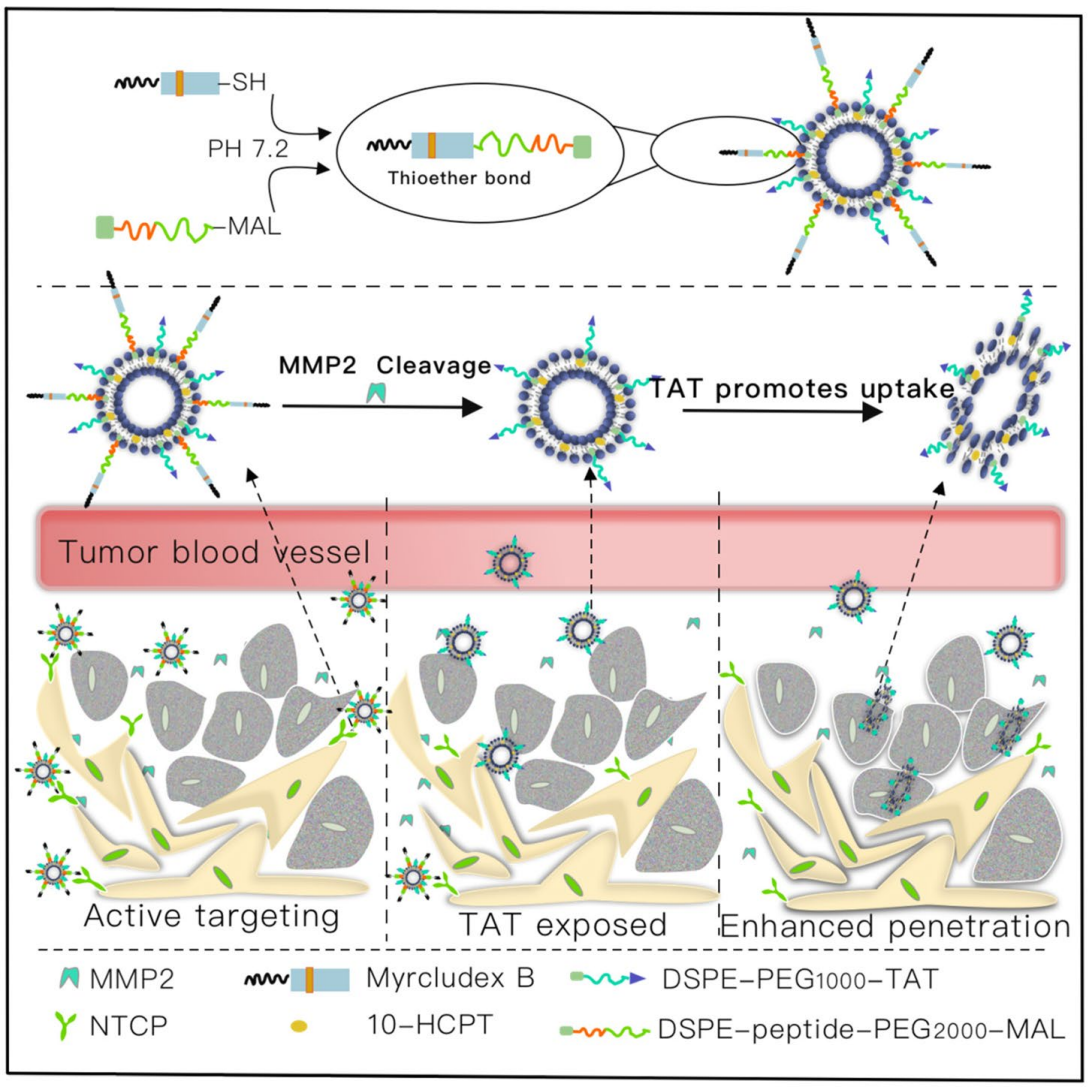
Transport HCPT to cancer cells by the myrB, TATp and MMP2-cleavable peptides co-modified liposomes for targeting therapy. I: MyrB-mediated liver targeting; II: MMP2 promotes PEG shedding and TATp exposure; III: TATp mediates increased cellular uptake.

In the present study, we designed a novel multifunctional myrB peptide, TAT peptide and MMP2-cleavable peptide co-modified liposomes (PPP-LIPs). The nanocarrier reaches the liver via a specific interaction between myrB and NTCP receptor. The MMP2-sensitive peptide linker is cut off by increased MMP2, and the PEG layer separates from the nanocarrier. Subsequently, previous hidden TATp exposure resulted in enhanced tumor targetability and internalization (Figure 1).

## 2. Materials and methods

### 2.1. Materials and reagents

Aladdin Industrial Corporation (Shanghai, China) provided hydroxycamptothecin (HCPT, purity > 98%), soybean phospholipids (SPC, purity > 98%), 1,2-dioloyl-sn-glycero-3-phosphoethanolamine (DOPE), Mal-PEG2k-NHS, cholesterol (Chol, purity > 95%), and PBS buffer. Guangzhou Tanshui Technology Co., Ltd supplied the DSPE-PEG1k-TAT and DSPE-PEG1k. Glbiochem (Shanghai, China) produced MMP2-cleavable peptides (Gly-Pro-Leu-Gly-Ile-Ala-Gly-Gln) and myrcludex B peptides (myrB, HBV preS/12-47myr). Novoprotein (Shanghai, China) provided the human active MMP2 protein. Beyotime Biotech Inc. (Shanghai, China) supplied Hoechst 33258, and Absin Bioscience Inc. (Shanghai, China) provided an apoptosis assay kit (Annexin V-FICT). The hydrochloride of N-Ethyl-N'-(3-dimethylaminopropyl) carbodiimide (EDC) was obtained from Aladdin Reagent Database Inc. AAT Bioquest Co. Ltd (Shanghai, China) supplied the TRITC-phalloidin. Sigma (Taufkirchen, Germany) delivered the 3-(4,5-dimethylthiazol-2-yl)-2,5-diphenyltetrazolium bromide (MTT). All other reagents were analytical grade, and all experiments were conducted using distilled water.

Hunan Silaikejingda Experimental Animal Co., Ltd. (Changsha, China) delivered the ICR rats (Certificate No. SCXK 2016-0002). GemPharmatech (Nanjing, China) provided BALB/c-nude mice, and Gibco (Mulgrave, Australia) supplied the fetal bovine serum (FBS), Certificate No. SCXK-2018-0008. All animal experiments were carried out following the Guilin Medical University Guidelines for Animal Experimentation, and the institution’s Animal Ethics Committee approved the protocol. The protocol also complied with the UK Animals (Scientific Procedures) Act, 1986 and associated guidelines and EU Directive 2010/63/EU for experimental animals.

### 2.2. Preparation of MMP2-responsive liposomes and surface modification of MMP2-responsive liposomes with myrB

#### 2.2.1. Preparation of MMP2-responsive HCPT-loaded liposomes

**Figure 2.**
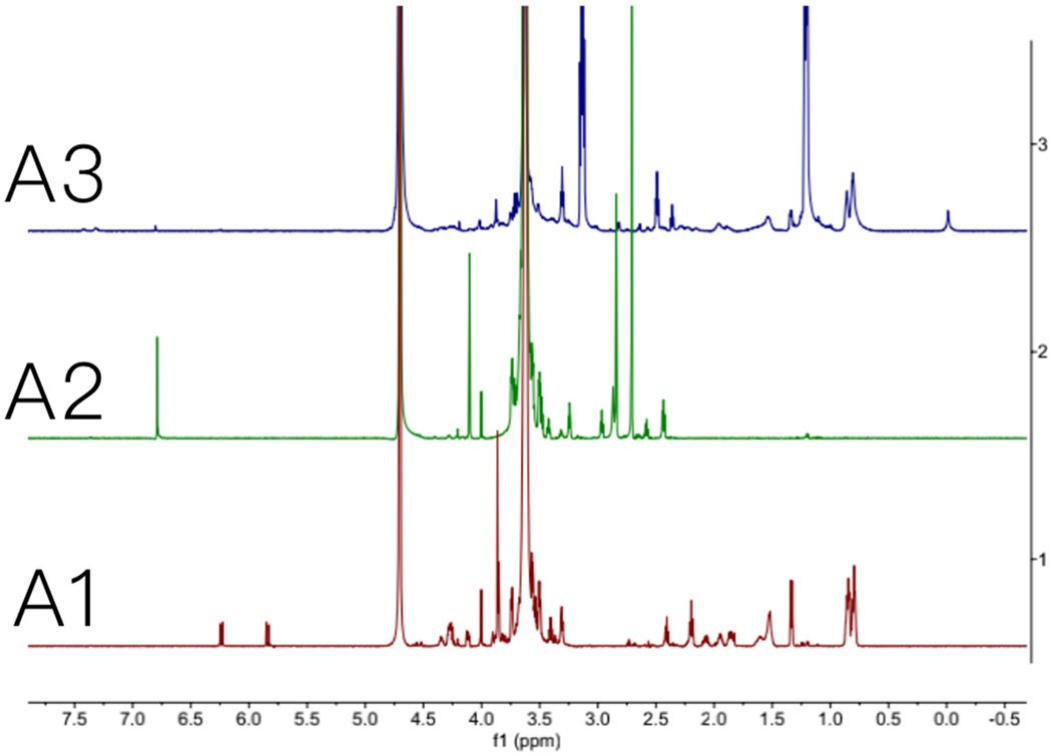
Characterization of MAL-PEG_2000_-NHS, MAL-PEG_2000_-peptide, and MAL-PEG_2000_-peptide-DOPE. (A1) ^1^H NMR of MAL-PEG_2000_-peptide; (A2) ^1^H NMR of MAL-PEG_2000_-NHS; (A3) ^1^H NMR of MAL-PEG_2000_-peptide-DOPE.

A previously published approach was used to synthesize Mal-PEG2k-PP-DOPE (Zhu et al., 2012; 2014). The MMP2 cleavable peptide’s primary amine was first reacted with the NHS group of Mal-PEG2k-NHS to form an amide bond, then the reaction product was conjugated with DOPE in the presence of EDC and NHS, and finally Mal-PEG2k-PP-DOPE was obtained. The ^1^H NMR spectra of Mal-PEG2k-PP, Mal-PEG2k-NHS and the final product, Mal-PEG2k-PP-DOPE, are shown in Figure 2A1, A2 and A3, respectively.

**Figure 3.**
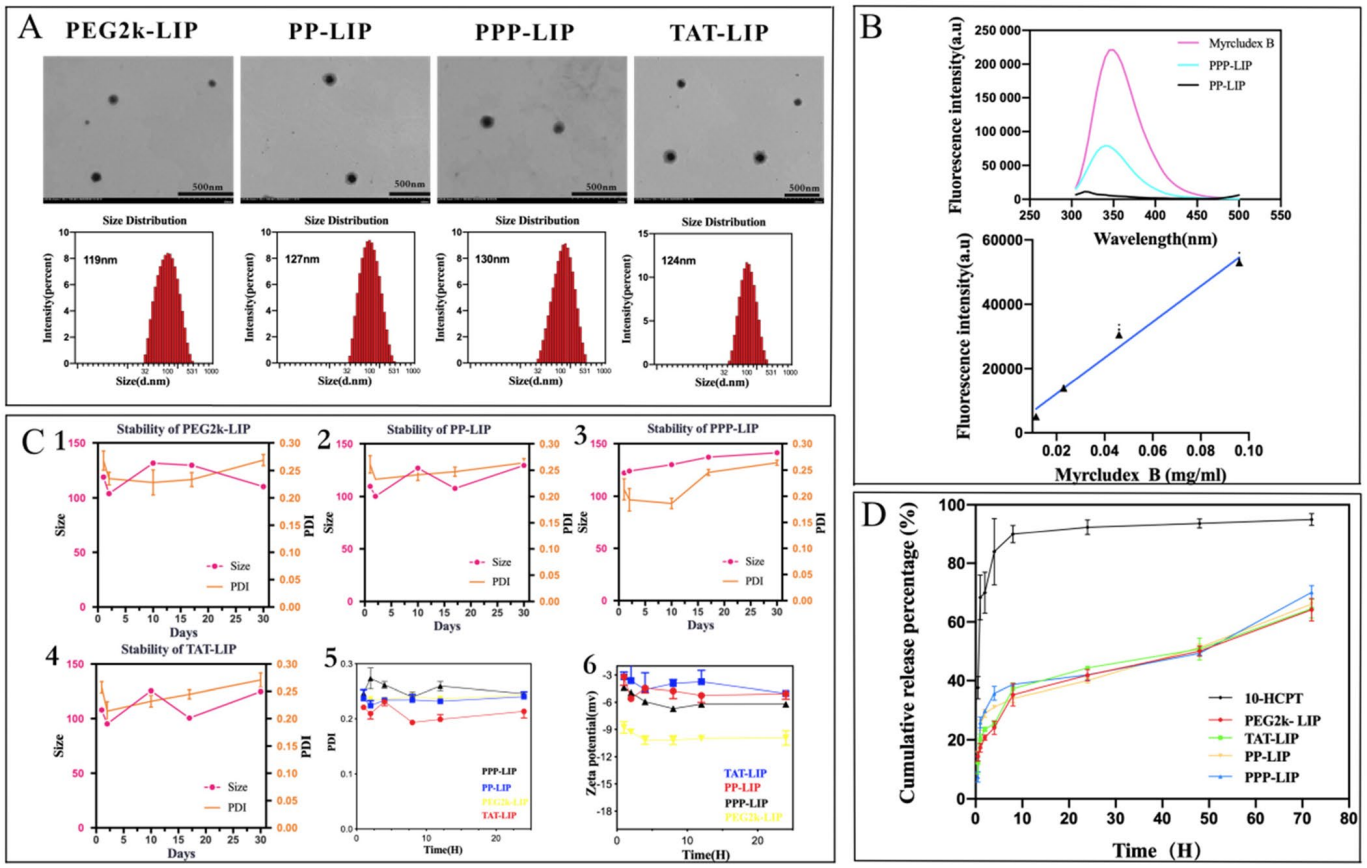
Characterization of liposomes. (A) the morphology and size distribution of liposomes. (B) fluorescence spectrum of myrB and liposomes. (C) the stability, PDI and zeta potential of liposomes. (D) *in vitro* release behavior of liposomes.

The lipid film hydration approach was used to make MMP2-responsive HCPT-loaded liposomes (PP-LIP) (Zhou et al., 2020). SPC, Chol, HCPT, DSPE-PEG1k-TAT, Mal-PEG2k-PP-DOPE, and DSPE-PEG1k were dissolved in 10 mL chloroform (at a ratio of 120:20:7:2:2:1), and a thin lipid film was generated after extracting the chloroform in a rotary evaporator at 50 °C and drying under vacuum for 2 hours. The lipid film was hydrated for 2 hours at room temperature with 5 mL of 10 mM HEPES buffer (pH 6.8). The lipid dispersions were sonicated for 5 minutes at 300 W in an ice bath using an ultrasonic cell disruptor (SCIENTZ-IID, Scientz, Ningbo, China) and then passed through a 200 nm polycarbonate membrane 10 times using a liposome extruder (LE-1, Morgec, Shanghai, China). SPC, Chol, HCTP, DSPE-PEG1k-TAT, and DSPE-PEG1k (at a ratio of 120:20:7:2:1) were utilized to produce lipid films for DSPE-PEG1k-TAT-modified liposomes (TAT-LIP). SPC, Chol, HCPT, DSPE-PEG1k, DSPE-PEG2k, and DSPE-PEG1k-TAT (at a ratio of 120:20:7:1:2:2) were utilized to produce lipid films for mPEG2k-modified liposomes (PEG2k-LIP). DiD- or Ce6-labelled liposomes were made the same way HCPT-loaded liposomes were made, except that DiD or Ce6 was used instead of HCPT.

#### 2.2.2. Surface modification of MMP2-responsive liposomes with myrB

A maleimide-thiol coupling procedure at room temperature for 4 hours produced myrB-modified MMP2-responsive liposomes (PPP-LIP). In a PBS solution, myrB was reacted with maleimide, which was changed on the surface of PP-LIP in a 3:1 ratio (pH 7.4). Unconjugated myrB was removed using a Sepharose CL4B column (Solarbio, Beijing, China).

#### 2.2.3. Characterization of myrB immobilization on liposomes

The maleimide-thiol coupling reaction was expected to immobilize PP-LIP via Mal-PEG by the myrB peptides. A fluorescence spectrometer (LabSolutions RF) was employed to confirm the peptide’s conjugation. After purification, the fluorescence intensity of PPP-LIP was measured using an emission wavelength of 350 nm and an excitation wavelength of 280 nm. A series of peptide solutions with varying concentrations (from 0.0115 to 0.092 mg/mL) in PBS (pH 7.4) were also detected under the same circumstances to create the standard curve. As a result, the conjugated peptide could be quantified.

#### 2.2.4. Particle size, zeta potential, and morphology of liposomes

The particle size, PDI, and zeta potential of various liposomes at 0.012 mg/mL HCPT were assessed using a Zetasizer (Nano-ZS90, Malvern Instruments, Malvern, UK). Transmission electron microscopy (TEM, HT7700, Hitachi, Japan) was used to observe the morphologies following staining with a 2% sodium phosphotungstate solution containing the same concentration of HCPT.

#### 2.2.5. Encapsulation efficiency and drug loading

For determining the encapsulation efficiency (EE) and drug loading (DL), free HCPT was isolated from liposomes using a Sepharose CL-4B gel column (DL). To summarize, 0.2 mL liposomes were placed onto a Sepharose CL-4B gel column and rinsed with HEPES buffer (pH 6.8), accompanied by the separation of liposomes and free drug. The drug entrapped was assessed by disrupting the liposome fraction with methanol. The concentration of HCPT was determined using reversed-phase InertSustain C18 analytical columns (250 mmx4.6 mm, 5 μm) with high-performance liquid chromatography (HPLC) (20 A, Shimadzu, Japan) set to 370 nm. The mobile phase was a 55:45 (*v/v*) combination of methanol and water. 1 mL/min was the flow rate. The injection volume was set to 20 μL. Liposomes’ EE and DL were computed using the following equations:

$$\mathrm{EE}\;(\%)=\frac{\text{weight of HCPT encapsulated in liposomes}}{\text{total weight of HCPT added}}x\;100\%$$

and,

$$\mathrm{DL}\;(\%)=\frac{\text{weight of HCPT encapsulated in liposomes}}{\left(\text{weight of HCPT encapsulated in liposomes})\;+(\text{weight of carrier materials}\right)}\times 100\%$$

#### 2.2.6. In vitro drug release

The dialysis approach was used to evaluate the *in vitro* release of the drug from drug-loaded liposomes. First, 0.1 mL of liposomes containing HCPT or free HCPT solutions were put into dialysis bags with an 8-14 kDa molecular weight cutoff. The dialysis bags were then immersed in 2 L PBS (pH 7.4) containing 0.05% Tween 80 at 37 °C with continual shaking at 150 rpm. The liposomes remaining in the dialysis belt were dissolved ultrasonically in methanol at predefined time intervals (0.5, 1, 2, 4, 8, 12, 24, 48, and 72 hours), and HPLC measured the quantities of HCPT.

$$\text{Drug release }(\%)=\frac{1-\text{HCPT content after dialysis}}{\text{HCPT content before dialysis}}\times \;100\%$$

#### 2.2.7. Stability study

Liposome stability under *in vitro* storage conditions is an important criterion for both *in vitro* and *in vivo* biomedical applications. The stability of liposomes was evaluated 1, 2, 4, 8, 12 and 24 days after preparation and storage at 4 °C. In addition, the 24 hours stability of a series of liposomes in FBS was measured at predetermined times (1, 2, 4, 8, 12 and 24 hours).

### 2.3. Cell culture

This study used LO2 and SMMC-7721 cells. The ChunMai Biotechnology Co. (Shanghai, China) supplied the LO2 cells; while, iCell Bioscience Inc. (Shanghai, China) provided the SMMC-7721 cells. At 37 °C with 5% CO2, LO2, and SMMC-7721, cells were cultured in RPMI-1640 media with 10% FBS. Before experiments, cells were precultured to 80% confluence.

### 2.4. Western blot

MMP2 and NTCP were detected utilizing Western blots. After two washes with PBS, RIPA lysis buffer with 1 mM PMSF was used to collect LO2 and SMMC-7721 cells. These were collected and mixed with protein loading buffer after a 20-minute, 12,000 rpm centrifugation at 4 °C. A nucleic acid trace detector measured total protein quantities (Thermo Forma, USA). A protein transfer machine separated 25 micrograms of protein samples using 10% SDS–PAGE (Bio-Rad, USA). MMP2 (1:2000) or NTCP (1:1000) primary antibodies were incubated overnight at 4 °C on the membranes after blocking for two hours at room temperature. TBST was used to clean the membranes, and then anti-mouse secondary antibodies were incubated with the clean membranes (1:4000). ECL prime Western blot detection reagent (GE Health care, Buckinghamshire, UK) and a ChemiDoc system (Bio-Rad, USA) were used to visualize MMP2 and NTCP.

### 2.5. Cytotoxicity assay

MTT assay was used to determine the cytotoxicity of HCPT injection, PEG2k-LIP, TAT-LIP, PP-LIP, and PPP-LIP. Firstly, the cells were planted (∼1 × 10^4^ cells per well) on a 96-well plate and incubated for 12 hours. Second, cells were treated for 48 hours with 100 μL fresh media containing various doses of HCPT (0.01, 0.1, 1, 10, and 20 μg/mL), whereas untreated cells in the same plate served as controls. Following that, 10 μL of MTT at a 5 mg/mL concentration in PBS was added to each well, and the cells were incubated for an additional 4 hours at 37 °C in 5% CO_2_. For dissolving the MTT formazan crystals, the medium was changed with 150 μL DMSO. The optical density at 490 (OD490) nm was then determined using a microplate reader (iMark, Bio-Rad, USA), and the half-maximal inhibitory concentration (IC50) values were computed using GraphPad software (Prism 8, GraphPad Software Inc, USA).

The cell viability ratio was estimated using the equation below:

$$\text{Cell viability ratio }(\%)=\frac{\mathrm{OD}490\;(\text{Drug group})}{\mathrm{OD}490\;(\text{Control group})}x\;100\%$$

### 2.6. Cellular uptake assay

LO2 and SMMC-7721 cells were plated onto coverglass in 24-well plates at a density of 5 × 10^4^ cells/well and allowed to adhere for 12 hours before performing confocal laser scanning microscopy (CLSM) investigations (LSM 710, Zeiss, Germany). Then, during 4 hours, 2 μg/mL nanoprobes (PEG2k-LIP, TAT-LIP, PP-LIP, and PPP-LIP; 2 μg/mL Ce6 equiv) were coincubated. To investigate the effect of MMP2 on cellular absorption, MMP2 was introduced to a 24-well plate in a preceding manner after coincubating PPP-LIP with MMP2 for 12 hours *in vitro* (PPP-LIP + M). The cells were then washed three times with sufficient prechilled PBS before being fixed in 4% paraformaldehyde for 30 minutes at 4 °C. The nuclei of the cells were stained with Hoechst 33258 for 5 minutes at room temperature. ZEN (Blue Edition) software was used to analyze the data.

LO2 and SMMC-7721 cells (10 × 10^4^ cells/well in 6-well plates) were grown in media for 12 hours before FACS analysis (BD FACSCanto plus). The medium was then withdrawn, the cells were washed three times with PBS, and the culture medium was replaced for 4 hours with fresh medium containing liposomes (at the same concentrations used for confocal fluorescence imaging investigations). The cells were then trypsinized and collected using 5-minute centrifugation at 1500 rpm. After washing with ice-cold PBS, the cells were resuspended in 500 mL buffer for flow cytometry experiments. The data (10,000 cell counts) were gathered and analyzed using the FlowJo v10 program.

### 2.7. Apoptosis assay

The Annexin V-FITC/PI apoptosis detection kit (Absin Bioscience, Shanghai, China) was employed according to the manufacturer’s protocol. LO2 and SMMC-7721 cells were plated in 6-well plates at a 1.5 × 10^6^ cell/well concentration. When cells reached the logarithmic stage, they were treated with 2 mL liposomes or 10 mL HCPT injection (using the same medication as for the MTT experiment) at a concentration of 1 μg/mL. The cells were trypsinized (without EDTA) and rinsed three times with PBS after 48 hours. The cells were then resuspended in 300 μL of 1 × binding buffer and stained with 5 μL of Annexin V-FITC and 5 μL of 100 μg/mL PI (dark operation). After 15 minutes of incubation, each resuspension solution received 200 μL of fresh 1 × binding buffer, and the cells were examined using flow cytometry and the software FlowJo.

### 2.8. Three-dimensional (3D) multicellular tumor spheroid (MCTS) inhibition experiment

Refer to the preceding procedure for spheroid creation. 200 μL/well of cell suspensions at optimal densities (0.5 × 10^4^ cells/mL for SMMC-7721 cells) were dispensed into 96-well plates after adding 40 μL of 1.5% agarose (low melting gel) aqueous solution (Solarbio, Beijing, China). The 96-well plates were then centrifuged at 1500 rpm for 10 minutes at 4 °C before being incubated at 37 °C with 5% CO_2_ and 95% humidity. An MCTS of around 500 μm in diameter was obtained after three days of incubation in 3 D multicellular tumor spheroids. For the liposomes or HCPT injection, a concentration of 2 μg/mL HCPT was administered to the MCTS. An inverted microscope (CKX53, Olympus Corporation, Japan) was used to measure the spheroid volumes of each group on different days and at predefined intervals (3, 5, 7, 9, and 11 days). It was determined that V = 4/3πr^3^ by using the tumor spheroid’s radius.

### 2.9. Near-infrared fluorescence imaging

Previously described *ex vivo* studies of liposomes in major organs were conducted. PP-LIP (0.2 mg/kg DiD) or PPP-LIP (0.2 mg/kg DiD) were injected into the tail vein of ICR mice in two groups of nine mice each. The mice were killed by cervical dislocation 2, 4, and 8 hours after treatment. After formaldehyde perfusion, key organs were removed and scanned using *in vivo* imaging equipment (UVP iBox Scientia). Through the semiquantitative analysis of *ex vivo* fluorescent pictures, the statistical graph of fluorescence (Jena analytical instruments AG) intensity for different organs was created.

### 2.10. *In vivo* tumor targeting evaluation

#### 2.10.1. Tumor implantation

One of the methods used to create the BALB/c nude mice model was based on earlier research. SMMC-7721 cells were diluted to 2.5 × 10^7^ cells/mL in RPMI-1640 medium before adding to the suspension. 0.2 mL of cell solution was then administered subcutaneously into BALB/c-nude mice (20–22 g) for tumor implantation. Additional studies were initiated when the tumors had grown to around ∼100 mm^3^ in diameter.

#### 2.10.2. In vivo imaging

The fluorescence pictures (630/20 nm excitation and 660/30 nm emission) were taken by an IVIS^®^ Lumina XR Series III imaging system (PerkinElmer, Hopkinton, USA) at various time intervals (1, 2, 4, 8, 12, and 24 h). It was also used to obtain fluorescence images of excised tumors and organs (heart, liver, kidney, lung, and spleen) using the same conditions as those previously indicated. IVIS^®^ Lumina XR living imaging software measured the tumor and organ fluorescence intensity.

#### 2.10.3. Antitumour efficacy

Tumor-bearing mice were randomly divided into five groups with four mice in each group. PBS was supplied to the control group of mice, each of the other four groups received a tail vein injection of 1 mg/kg of HCPT, PEG2k-LIP, PP-LIP, and PPP-LIP on days 0, 2, 4, 6, 8, 10, and 12. Every two days, body weight and tumor volume were recorded, the tumor volume was estimated using the formula: volume = (length × width^2^)/2. Tumors, livers, kidneys, lungs, spleens, and hearts were collected from mice euthanized on day 14. After weighing the tumors, the tissues were preserved in 4% neutral buffered formalin for two days. Then, using the normal methodology, hematoxylin, and eosin (H&E) staining was used to monitor the morphological features of each organ. Finally, tumor tissue was stained with TUNEL to study tumor apoptosis in the various treatment groups.

### 2.11. Statistical analysis

The student’s t-test was used to compare groups based on the mean ± standard deviation (SD) data. If a *p*-value falls below the thresholds for statistical significance (*p < .05, **p < .01, and ***p < .001), it was considered statistically significant.

## 3. Results and discussion

### 3.1. Characterization of liposomes

#### 3.1.1. Morphology and particle size distribution of liposomes

The morphology and size distribution of liposomes are displayed in Figure 3A, the liposome diameter was consistent with its spherical form and good dispersion. The average diameter was about 125 nanometers, PDI less than 0.30 (Table 1), liposomes treated with myrB had somewhat larger particle sizes than unmodified liposomes. The negative charge of myrB-lipopeptides kept the zeta potential of nanoparticles negative, which avoided particle sequestration in the lungs or quick clearance by cells expressing scavenger receptors (Somiya et al., 2016). The encapsulation efficiency of HCPT was consistently about 85%, indicating that the medication was well absorbed into the liposome.

**Table 1.** The average particle size, PDI, zeta potential and encapsulation efficiency of liposomes.

	Size (nm)	PDI	Zeta potential (mv)	Encapsulation efficiency (%)
PEG2k-LIP	119.3 ± 6.1	0.243±0.041	−9.8±1.3	88.5±2.6
PP-LIP	127.1 ± 6.5	0.254±0.024	−4.3±1.7	84.6±1.8
PPP-LIP	130.2 ± 4.3	0.220±0.049	−5.7±1.2	83.2±1.6
TAT-LIP	124.7 ± 5.3	0.239±0.045	−4.7±1.9	85.7±2.2

Surface properties, in particular, are a significant feature of nanoparticles. To avoid particle sequestration in the lungs (due to a positive charge) or quick clearance by cells expressing scavenger receptors, the surface charge (zeta potential) should be somewhat negative. Steric stabilization by PEGylation mediates long-circulating characteristics and inhibits opsonization in addition to surface charge. Liposomes’ size distribution is another essential feature. Since the effective size for drug delivery systems should be less than 200 nm, the threshold vesicle size for extravasation into a tumor’s extracellular space was discovered to be around 400 nm (Peer et al., 2007). As a result, it appears that the nanoparticles we created are ideal for drug delivery to tumors.

#### 3.1.2. Determination of surface modification content of myrcludex B

PPP-LIP was created in this study by directly conjugating myrB to the surface of premade liposomes, unconjugated myrB was removed by size-exclusion chromatography (SEC). We observed that myrB contained tryptophan, which we were able to quantify using fluorescence spectroscopy at 350 nm with an excitation spectrum of 280 nm, allowing us to calculate the grafting ratio (Yang et al., 2016). The amount of myrB bound to the surface of liposomes was assessed and the peptide content in PPP-LIP was around 37.2 + 2.4 g/mg lipids (Figure 3B).

#### 3.1.3. Stability study of liposomes

The stability of liposomes *in vitro* is a critical criterion for *in vitro* and *in vivo* biomedical applications. Additionally, the liposomes should remain stable in the bloodstream. The liposomes dispersed easily after being stored at 4 °C for one month, and their average size and PDI were quite stable during storage (Figure 3C). The liposomes’ serum stability *in vitro* revealed that the liposomes were stable during blood circulation.

#### 3.1.4. In vitro release of liposomes

In contrast to the quick release of free HCPT, all liposomes demonstrated continuous release with no burst release (Figure 3D). Liposomes had a cumulative release rate of less than 40% after 12 hours, but free HCPT had a cumulative release rate of more than 90%. There were no significant changes in drug release among all liposomes at any phase, indicating that the surface modification of nanoparticles had no effect.

### 3.2. Western blot assay

**Figure 4.**
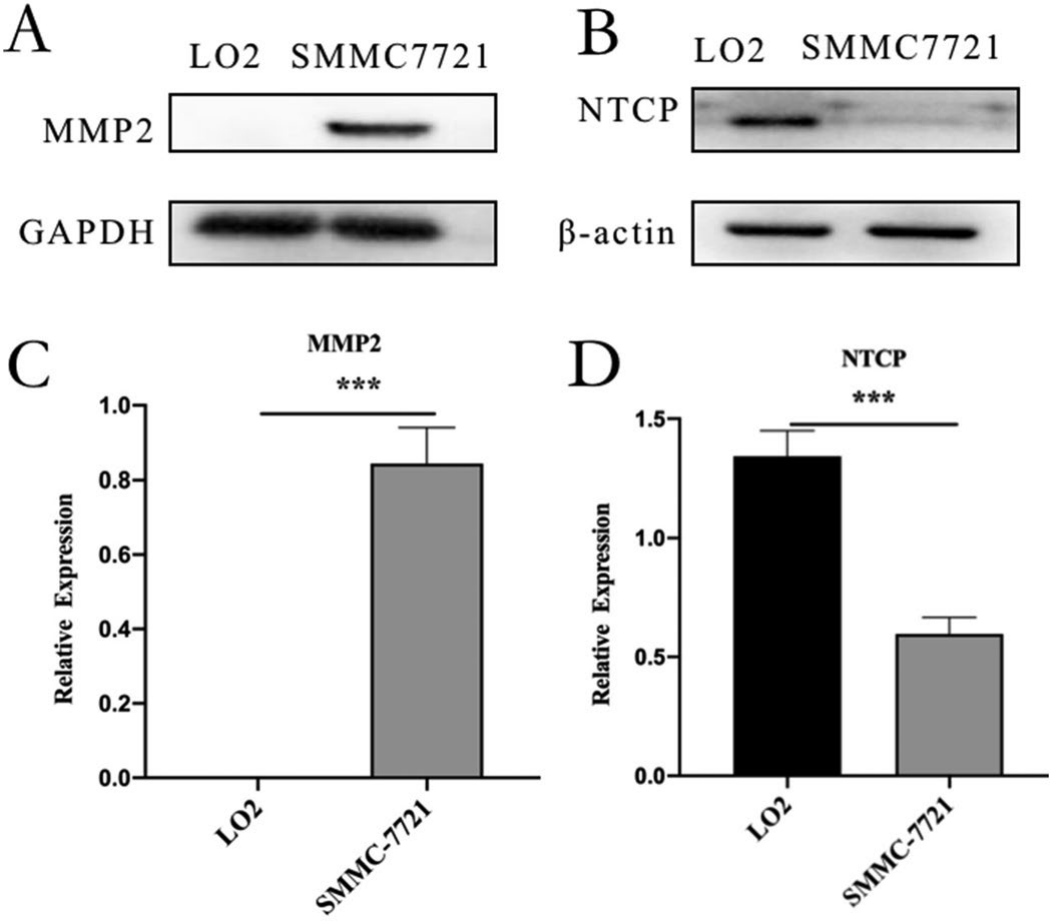
Western blot analysis: (A) western blot analysis of MMP2 in LO2 and SMMC-7721 cells. (B) western blot analysis of NTCP in LO2 and SMMC-7721 cells. (C) quantitative analysis of MMP2 western blot assay. (D) quantitative analysis of NTCP Western blot assay. Data represent mean ± standard deviation (*n* = 3). ***p < .001, *p < .05.

SMMC-7721 cells overexpressed MMP2, and LO2 cells overexpressed NTCP (Figure 4A and B). MMP2 was substantially expressed in SMMC-7721 cells but was expressed at low levels or not at all in LO2 cells, NTCP expression was substantially higher in LO2 cells than in SMMC-7721 cells. The expression difference of NTCP or MMP2 on LO2 and SMMC-7721 cells supported the follow-up experiment.

### 3.3. Cell uptake assay

**Figure 5.**
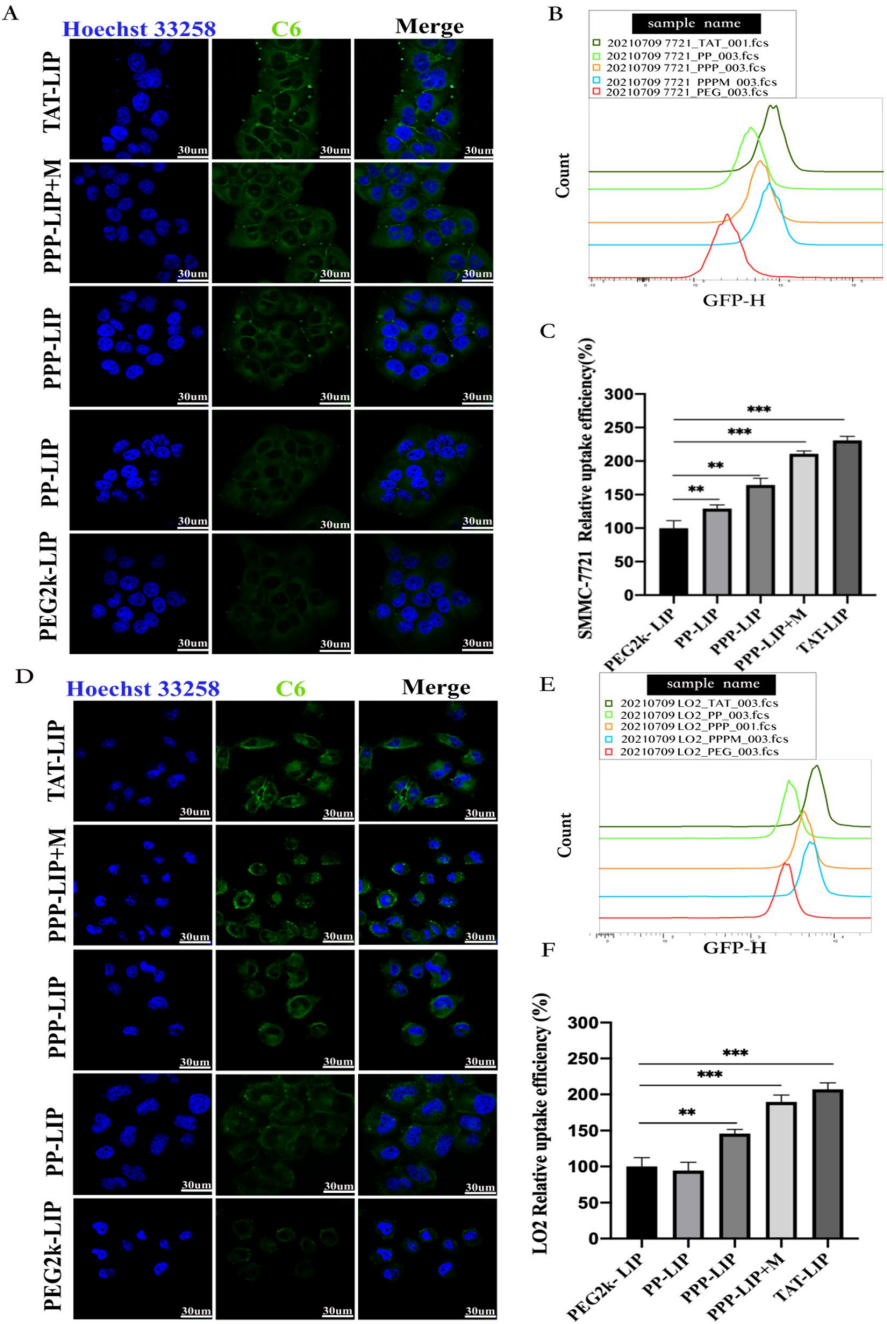
Cellular uptake and localization: (A) confocal images of SMMC-7721 cells exposured to TAT-LIP, PPP-LIP, PP-LIP, PEG2k-LIP and PPP-LIP + M for 4 hours, respectively. (B) flow cytometry analysis of SMMC-7721 cellular uptake for 4 hours. (C) quantitative analysis of flow cytometry SMMC-7721 uptake. (D) confocal images of LO2 cells exposured to TAT-LIP, PPP-LIP, PP-LIP, PEG2k-LIP and PPP-LIP + M for 4 hours, respectively. (E) flow cytometry analysis of LO2 cellular uptake for 4 hours. (F) quantitative analysis of flow cytometry LO2 uptake. Data represent mean ± standard deviation (*n* = 3). ***p < .001, *p < .05.

Ce6 was located in LO2 and SMMC-7721 cells using green fluorescence, and cell nuclei were recognized using blue fluorescence and Hoechst dye. Ce6 was mostly found in the cytoplasm following endocytosis (Figure 5A and D). LO2 and SMMC-7721 cells treated with Ce6-loaded PEG2k-LIP for 4 hours showed only a faint green fluorescence, indicating that only a modest amount of Ce6 had reached the cells. One explanation for this is that PEG2k-LIP exhibited a greater negative potential, increasing electrostatic repulsion, and the presence of long-chain PEG inhibited cell internalization (Mickler et al., 2011; Vila-Caballer et al., 2016). Ce6 fluorescence distribution in TAT-LIP-treated cells was always higher than in those treated with other liposomes. It was found that, under identical conditions, the mean fluorescence levels of SMMC-7721 cells treated with PP-LIP and PPP-LIP were substantially higher than those of cells treated with PEG2k-LIP. Greater Ce6 fluorescence intensity was seen in the cells treated with PPP-LIP + M (liposomes had been preincubated with MMP2 for 12 hours) than those treated with PPP-LIP. PP-LIP-treated LO2 cells retained the same mean fluorescence level as cells treated with PEG2k-LIP (Figure 5E and F). It could be because the peptide linker was cleaved by extracellular MMP2 in the SMMC-7721 cell microenvironment, allowing the previously buried TATp to be exposed for cell internalization. Small animal *in vivo* imaging data shows that MMP2 was used as a stimulant and synergized with TAT to increase tumor targeting and internalization in our newly developed nanocarrier, consistent with these findings.

PEG2k-LIP, PP-LIP, PPP-LIP, PPP-LIP + M, and TAT-LIP internalization into SMMC-7721 and LO2 cells were further analyzed quantitatively using flow cytometry (FCM). The same protocols for CLSM therapy were employed before cell harvesting for FCM analysis. The mean fluorescence of SMMC-7721 cells treated with PP-LIP for 4 hours was substantially greater than that of PEG2k-LIP cells treated under the same conditions (*p <* .05) (Figure 5B and C). For 4 hours, LO2 cells were treated with either PP-LIP or PEG2k-LIP and the mean relative Ce6 fluorescence was measured (Figure 5E and F). There is a possibility that the deprotection of PEG by the MMP2-overexpressing tumor cell microenvironment led to increased tumor penetration by exposing TATp in PP-LIP. It’s worth mentioning that LO2 cells treated with PPP-LIP had a higher fluorescence level than those treated with PP-LIP. The hepatocellular absorption of PPP-LIP could be attributed to ligand-receptor interactions. According to the literatures, the first 47 amino acids of HBV PreS1 protein were used to create a safe and effective completely synthesized peptide with the potential to target liver cells, HBV PreS/2–31-modified liposome nanoparticles could achieve the best balance between avoiding blood clearance and targeting ability, it was capable of delivering drugs to hepatocytes with great specificity *in vitro* and *in vivo* (Zhang et al., 2014; Zhang et al.,2015; Witzigmann et al., 2019). The SMMC-7721 cell median fluorescence values are shown in Figure 5C and were analyzed by FlowJo software. After 4 hours of incubation, we found a 30% fluorescence intensity rise in the PP-LIP-treated group compared to the PEG2k-LIP-treated group. Treatment with TAT-LIP or PPP-LIP + M increased C6 fluorescence by about twofold compared to treatment with PEG2k-LIP under the same circumstances. As a whole, our findings are in line with those obtained using the CLSM method.

### 3.4. Antitumour effect in vitro

A suitable nano-drug delivery system should be stable and have low toxicity *in vivo* and *in vitro*. First, blank liposomes (PP-LIP, PEG2k-LIP, PPP-LIP and TAT-LIP) were investigated in noncancerous LO2 cells and SMMC-7721 cells (Figure 6A and B). Even at a high dose of 400 μg/mL, no substantial suppression of cell proliferation was seen in any group (lipid membrane material). These findings revealed that our nanosystem was safe for cells due to its low toxicity. The lower cellular drug concentration in liposomes than in free HCPT solution may explain the reduced cytotoxicity of distinct liposome groups, as illustrated in Figure 6C and D. To overcome its nonspecific cytotoxicity, PEGylation could protect a positive charge in PEG2k-LIP, which resulted in much-decreased cytotoxicity. the IC50 values of PEG2k-LIP, PP-LIP, and PPP-LIP for SMMC-7721 cells were 15.18, 5.675 and 5.118 μg/mL, respectively, and 4.03, 2.927 and 1.743 μg/mL for LO2 cells. These results demonstrate that DOPE-peptide-PEG2k could be fractured by MMP2 overexpression in SMMC-7721 cells, causing PEG deprotection and resulting in TAT exposure, which enhanced tumor penetration subsequently effectively disrupted tumor cells. PPP-LIP showed higher cytotoxicity than PP-LIP in LO2 cells, which might be due to PPP-LIP being internalized in LO2 cells via NTCP-mediated endocytosis.

Figure 6.*In vitro* cellular toxicity: cytotoxicity of blank liposomes without HCPT-loaded in LO2 cells (A) and SMMC-7721 cells (B); cytotoxicity of HCPT-loaded liposomes in LO2 cells (C) and SMMC-7721 cells (D); flow apoptosis assay of HCPT-loaded liposomes in SMMC-7721 cells (E) and LO2 cells (G); statistical analysis of flow cytometry in SMMC-7721 cells (F) and LO2 cells (H); 3 D tumor ball inhibition experiment (I); spheriod volume of tumor globules at 11 days (J); Data represent mean ± standard deviation (*n* = 5). ***p < .001, *p < .05.

**Figure:**
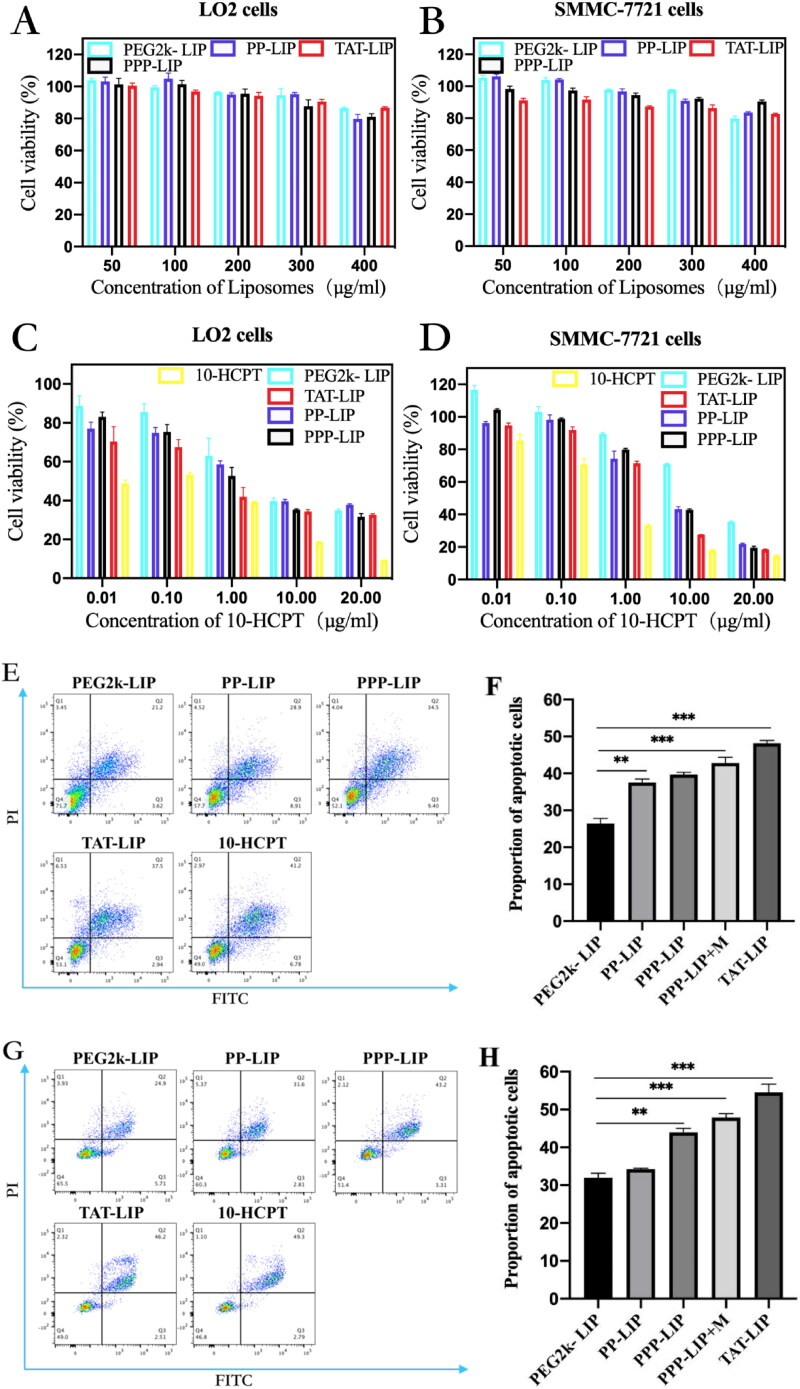

**Figure:**
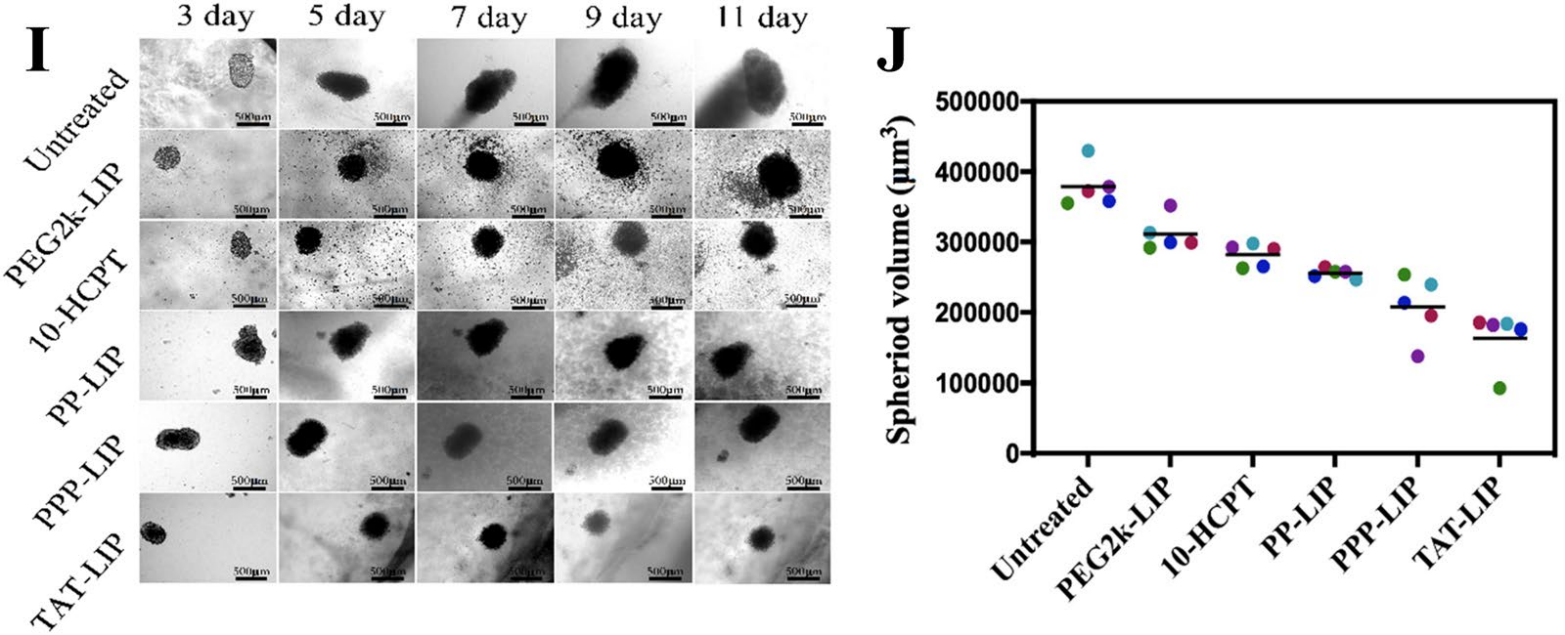

Double labeling with Annexin V-FITC and Propidium Iodide (PI) flow cytometry was used in an apoptosis experiment to examine cell death and necrosis after treatment with free HCPT and various liposomes (Lin et al., 2020; Figure 6E and G). PP-LIP (37.5%) increased apoptosis of SMMC-7721 cells compared to PEG2k-LIP (26.4%) (Figure 6F). In the LO2 cells, there were no significant cell apoptosis differences among PP-LIP (34.2%) and PEG2k-LIP (32.64%) (Figure 6H). To evaluate the stimuli-responsive nanoparticle drug delivery systems, an MCTS 3 D tumor model was established by SMMC-7721 tumor cells (Figure 6I). PP-LIP, PPP-LIP and TAT-LIP could relatively obviously inhibit the growth of 3D MCTSs. (Figure 6J). These findings suggested an outstanding synergy between TATp, MMP2-cleavable peptides, and liposomes for tumor cell death.

### 3.5. In vivo targeting evaluation

**Figure 7.**
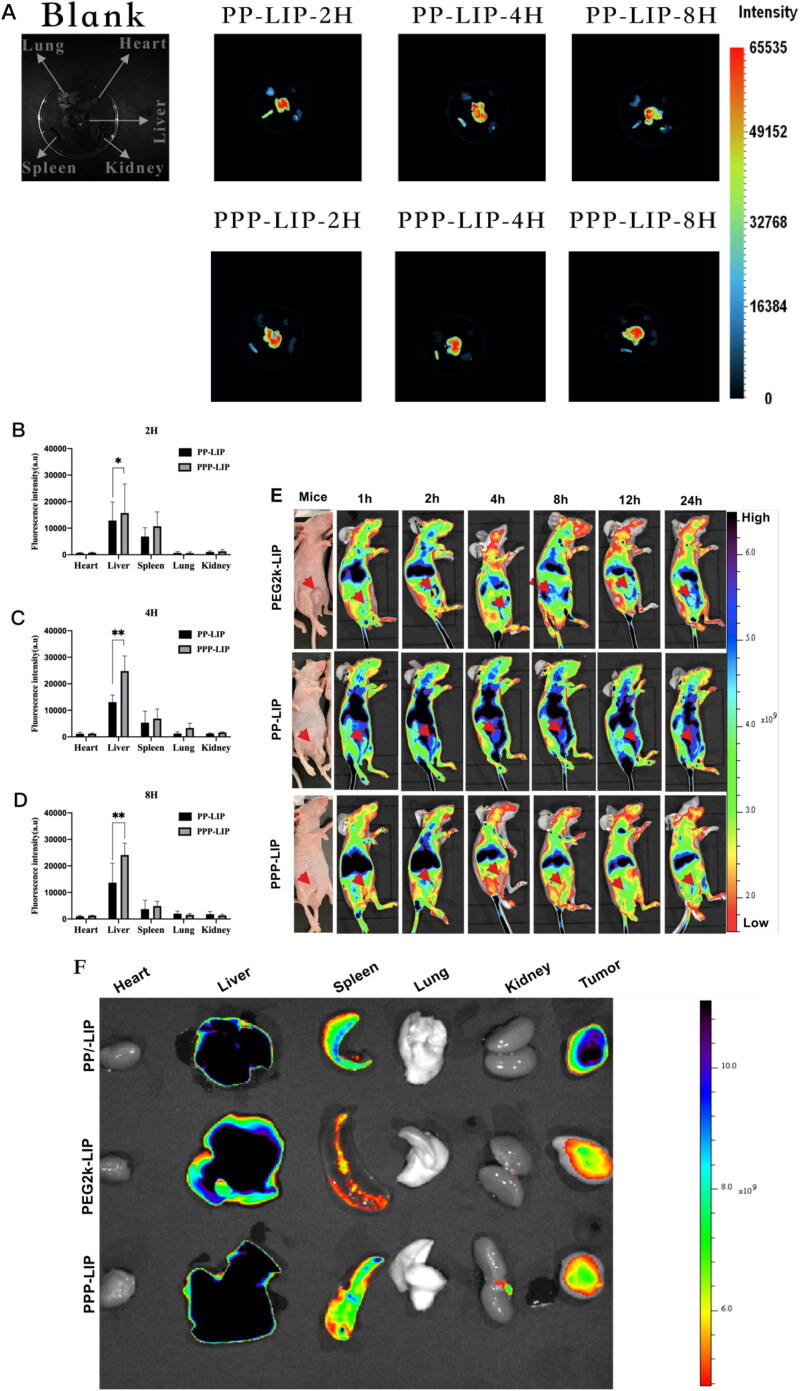
*Ex vivo* imaging of liposomes in major organs, *in vivo* biodistribution and *in vitro* fluorescence images of organs and tumors: (A) ICR mice were intravenously injected with DiD-loaded PP-LIP and PPP-LIP respectively. Organs were harvested 2, 4 and 8 hours after administration. The statistical graph of the fluorescence intensity of organs based on the semi-quantitative analysis to the *ex vivo* fluorescent images of ICR mice attained 2 hours (B), 4 hours (C), and 8 hours (D) after *i.v.* administration with DiD-labeled PP-LIP or PPP-LIP. (E) *In vivo* time-dependent whole body fluorescence imaging of SMMC-7721 tumor-bearing mice after intravenous injection of PEG2k-LIP, PP-LIP and PPP-LIP. (F) *In vitro* fluorescence images of major organs and tumors of mice after intravenous injection of PEG2k-LIP, PP-LIP and PPP-LIP over a period of 24 hours. Data represent mean ± standard deviation (*n* = 3). ***p < .001, *p < .05.

The *in vivo* liver targeting of PPP-LIP was evaluated by a near-infrared fluorescence imaging approach (Figure 7A). As anticipated, PPP-LIP accumulated primarily in the liver with a negligible distribution in other organs at various periods compared to PP-LIP. Semiquantitative analysis of the *ex vivo* fluorescent pictures (Figure 7B, C, and D) revealed that the fluorescence intensity in the PPP-LIP injection group’s livers was significantly greater than that in the PP-LIP injection group’s livers at various time points (*p* < .01). These results indicate that myrB was successfully changed on the liposome surface and that PPP-LIP efficiently delivers the photosensitizer DiD to the liver. Indeed, myrB's liver-targeted properties have been established in earlier animal and cell research, and a pharmacokinetic assessment of myrB in chimps, the most relevant animal model, revealed evidence of liver targeting. It was confirmed that a substantial dose of myrB subcutaneous injection resulted in at least 15 hours of target saturation of 80% (Schulze et al., 2010; Blank et al., 2016).

To investigate the *in vivo* tumor targetability of PEG2k-LIP, PP-LIP, and PPP-LIP, we examined the biodistribution of DiD-labeled PEG2k-LIP, PP-LIP, and PPP-LIP following intravenous treatment in SMMC-7721 tumor-bearing BALB/c-nude mice (Figure 7E). Clear fluorescence was observed in the liver location following PPP-LIP treatment, and the fluorescence signals gradually diminished over time while maintaining a reasonably high fluorescence level. Additionally, other organs (excluding the liver) had a lower fluorescence intensity, indicating that PPP-LIP was more effective at targeting the liver *in vivo*. On the other hand, when compared to the PPP-LIP- and PEG2k-LIP-treated animals, the PP-LIP-treated mice, showed a considerable increase in the fluorescence intensity signal of DiD at the tumor location. The significant tumor targeting ability could be the accelerated cell internalization produced by the peptide linker was cleaved by extracellular MMP2, exposing the previously hidden TATp.

*Ex vivo* imaging was also used to confirm the dispersion of the liposomes in tissues (i.e. tumor, heart, liver, kidney, lung and spleen) (Figure 7F). PP-LIP had the maximum fluorescence intensity in tumor areas, approximately 5-fold higher than PEG2k-LIP and 3-fold higher than PPP-LIP. Furthermore, other organs (save the liver) showed lesser fluorescence intensity, indicating that PP-LIP had improved *in vivo* tumor targeting. Similar to prior findings, fluorescence was primarily noticed in the liver rather than the tumor location for PPP-LIP.

### 3.6. In vivo antitumour efficiency

In terms of tumor growth, PP-LIP-treated mice had the slowest tumor growth rates, smallest tumor volumes, and lightest tumor weight (Figure 8), indicating that PP-LIP had a significant antitumor ability. This could be attributable to the passive tumor targeting of EPR effect and improvement of cell internalization following MMP2-activated exposure of previously hidden TATp. PPP-LIP might improve HCPT accumulation in the liver rather than at tumor sites. Furthermore, the body weights of mice treated with PPP-LIP, PP-LIP, and PEG2k-LIP were not substantially different from the control group, demonstrating that the nanomedicines were not hazardous to mice.

Figure 8.Tumor suppression study *in vivo* via intravenous injection under skin once every two days: (A) images of the tumors at the 13th day post-treatment. (B) body weight changes of the mice with different groups. (C) the relative tumor volume. (D) tumor weights after post-treatment in different groups (*n* = 4). (E) H&E staining images of tumor and major organ tissues in mice, which were sacrificed at the 13th day after treatment of various groups. (F) TUNEL staining images of tumor tissues in mice, which were sacrificed at the 13th day after treatment of various groups.

**Figure:**
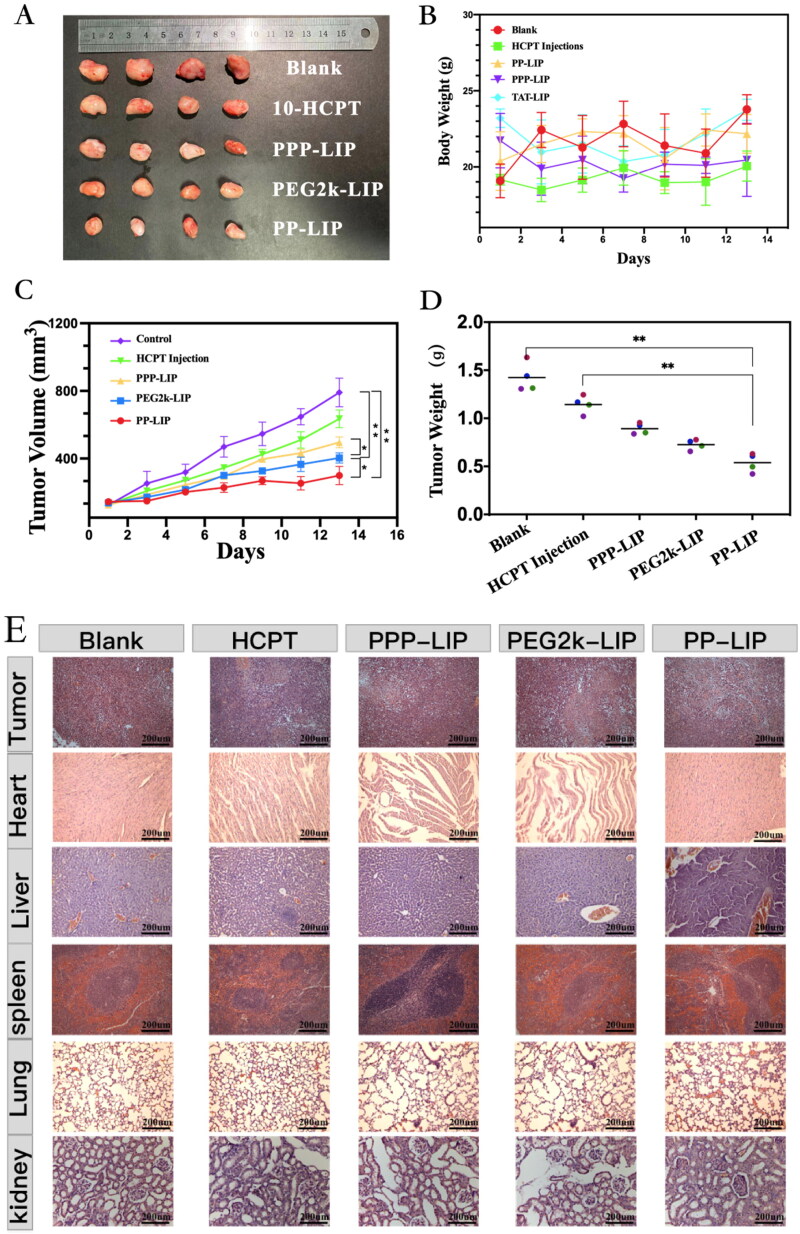

**Figure:**
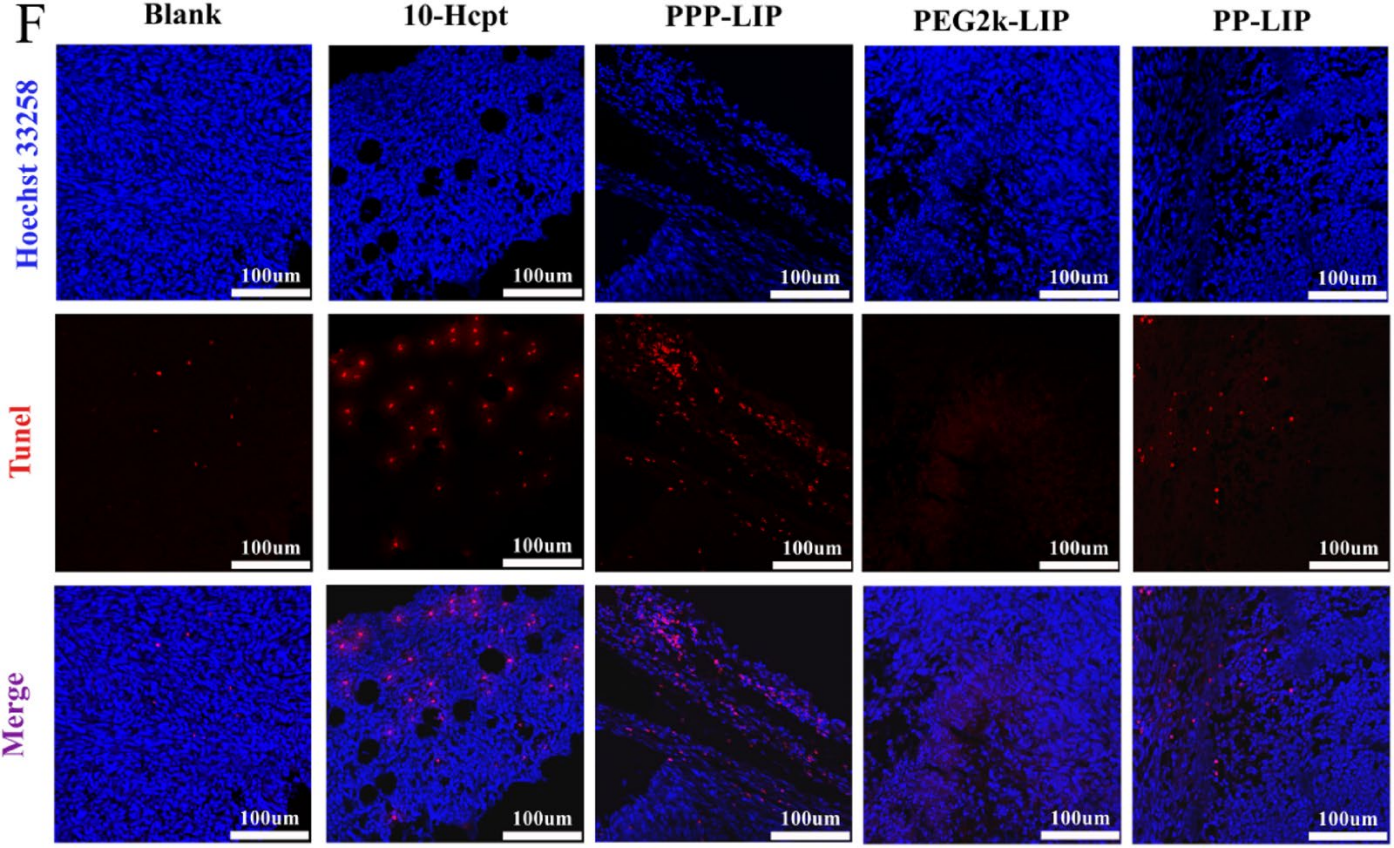

Histological investigations employing H&E staining demonstrated that PP-LIP induced widespread necrosis in the tumor sites while causing only minor damage to the liver and spleen (Figure 8E). TUNEL detection of tumor tissues extracted after treatment revealed that the PP-LIP therapy group had the most powerful therapeutic effects (Figure 8F). PP-LIP therapy group had the greatest red fluorescence, while the HCPT-treated group had only a few fluorescence signals, it is obvious that the antitumor activity of HCPT *in vivo* was significantly improved. Additionally, PPP-LIP modified with myrB demonstrated an exceptional ability to target the liver.

## 4. Conclusion

Novel myrB, TATp and MMP2 sensitive peptides co-modified nanoparticles for HCC targeted therapy were generated consistently in this investigation. The PPP-LIP and PP-LIP that were created had a high drug-loaded capability and great internal and exterior stability. *In vitro*, PPP-LIP and PP-LIP dramatically increased the cellular absorption of HCPT in SMMC-7721 cells with strong MMP2 expression, resulting in cancer cell death. PPP-LIP could readily accumulate in the liver of ICR mice and SMMC-7721 tumor-bearing animals due to the presence of myrB, according to *in vivo* alive imaging. Furthermore, PP-LIP demonstrated improved tumor targeting *in vivo*; eventually, the liposomes demonstrated considerably higher tumor growth inhibition and reduced systemic toxicity in a tumor-bearing mice model. Finally, this study presents a liposome drug delivery platform in which NTCP-mediated MMP2 induces TATp exposure to boost cellular absorption, indicating a tremendous potential for selective intracellular delivery of hydrophobic anticancer medicines in the liver tumor cells.
